# Chinese survey of candidasis in ICUs: China-SCAN study

**DOI:** 10.1186/cc9659

**Published:** 2011-03-11

**Authors:** HB Qiu

**Affiliations:** 1 Zhongda Hospital of Southeast University, Nanjing, China

## Introduction

This is the first national multicenter epidemiology study of invasive candida infections (ICIs) within ICUs in China. The objectives included describing the epidemiology, patient characteristics and management of these ICIs.

## Methods

The study used a prospective observational design. A total of 68 ICUs in China participated. The study was initiated on 1 November 2009 and will close on 30 April 2011. During the study period all consecutive patients above 18 years diagnosed as proven ICI after being admitted into the ICUs were eligible for enrollment. For each episode of ICI, demographic data, underlying diseases, severity of illness, risk factors, diagnosis, reported pathogen of fungal infection, process of treatment and survival at discharge were recorded. A total of 203 ICI cases were identified by the end of October 2010; since CRF collection and data management for part of the cases are ongoing, here we report the interim analysis results of 145 proven ICIs.

## Results

Among 145 eligible ICI patients, 134 (92.4%) had isolated candidemia, two (1.4%) had invasive candidiasis with candidemia, and nine (6.2%) had invasive candidiasis without documented candidemia. The median time ICI occurred was 9 days after ICU admission. The mean APACHE II was 26.6 at ICU admission (SD 7.2). The frequency of risk factors within 2 weeks before ICI were 107 patients (73.8%) with central venous catheterization, 117 (80.7%) with antibiotic therapy >5 days, 112 (77.2%) with invasive mechanical ventilation and 62 (42.8%) with total parenteral nutrition. The case fatality ratio of ICI in the ICU was 34.5% (50/145). A total of 156 isolates were collected, *C. albicans *accounted for 48.1% (75/156) of the isolates, followed by *C. parapsilosis *(14.1%), *C. tropicalis *(14.1%) and *C. glabrata *(9.6%). Seventy-five patients were reported with *C. albicans *infection (51.7%), among them five patients were reported as co-infected with other candida. Forty-three patients (29.7%) received initial antifungal therapy before or on the day of first positive sample drawn, 81 patients (55.9%) initiated therapy after the ICI diagnosis was proven. Initial treatment was mainly based on the use of a single antifungal agent (98.4%), and the treatment protocol was modified in 64 patients (44%) due to identification of causative Candida species, susceptibility reports or other reasons.

## Conclusions

In China more than 90% of ICIs in the ICU were diagnosed by candidemia. Non-albicans Candida species accounted for one-half of the Candida isolates. Mortality of ICIs in the ICU remains high; however, targeted therapy accounted for more than 50% of initial antifungal therapy.

